# Milk Fat Intake and Telomere Length in U.S. Women and Men: The Role of the Milk Fat Fraction

**DOI:** 10.1155/2019/1574021

**Published:** 2019-10-28

**Authors:** Larry A. Tucker

**Affiliations:** Brigham Young University, College of Life Sciences, 106 SFH, Provo, Utah 84602, USA

## Abstract

The associations between milk intake frequency and milk fat consumption and telomere length, an index of biological aging, were studied using an NHANES sample of 5,834 U.S. adults and a cross-sectional design. The milk consumption variables were assessed with the NHANES Diet Behavior and Nutrition questionnaire. The quantitative polymerase chain reaction method was used to measure leukocyte telomere length. Results showed that milk consumption frequency was not related to telomere length; however, there was a strong association between milk fat intake and telomere length. With the sample delimited to milk drinkers only, milk fat intake was linearly and inversely related to telomere length, after adjusting for the covariates (*F* = 8.6, *P* = 0.0066). For each 1 percentage point increase in milk fat consumed (e.g., 1% to 2%), adults had more than 4 years of additional biological aging. With milk fat intake divided into 5 categories (i.e., milk abstainers, nonfat, 1%, 2%, and full-fat milk), mean telomere lengths differed across the categories (*F* = 4.1, *P* = 0.0093). The mean telomere difference between the extremes of milk fat intake (nonfat vs. full-fat) was 145 base pairs, representing years of additional biological aging for full-fat milk consumers. Effect modification testing indicated that the milk fat and cellular aging association may be partly due to saturated fat intake differences across the milk fat groups. When the sample was delimited to adults reporting only high total saturated fat intake (tertile 3), the milk fat and telomere relationship was strong. However, when the sample was restricted to adults reporting only low saturated fat consumption (tertile 1), there was no relationship between milk fat intake and telomere length. Overall, the findings highlight an association of increased biological aging in U.S. adults who consumed high-fat milk. The results support the latest Dietary Guidelines for Americans (2015–2020), which recommend consumption of low-fat milk, but not high-fat milk, as part of a healthy diet.

## 1. Introduction

Investigations evaluating the effect of adult milk consumption on health and disease have produced inconsistent findings. Some studies indicate that the consumption of cow's milk promotes health, while others show that it increases risk of disease and mortality. Numerous investigations highlight the mixed results.

In a 2018 study by Feskanich et al. [1], milk intake was associated with a lower risk of hip fracture, whereas in a 2018 investigation by Michaelsson et al., milk consumption was linked to an increased risk of hip fracture [2]. Further, a meta-analysis that included six studies focusing on women and three of men concluded that milk intake and hip fracture are unrelated [3].

In a meta-analysis of 19 studies concentrating on colorectal cancer, Aune et al. [4] determined that milk intake reduces risk, whereas in an evaluation of 32 investigations, the same researcher [5] concluded that milk consumption increases risk of prostate cancer. Similarly, some research indicates that dairy intake reduces the risk of type II diabetes [6, 7], whereas other studies show that dairy consumption is linked to increased insulin resistance [8–10]. Several investigations have concluded that dairy intake is unrelated to type II diabetes and associated metabolic factors [11, 12]. Lastly, in a Japanese cohort, milk intake was inversely associated with all-cause mortality [13], but in a Swedish group, milk consumption was related to increased all-cause mortality [14].

Clearly, the effects of adult milk consumption on health and disease are varied, and in recent years, questions about the influence of milk have been further complicated with debate about the effect of milk fat on disease risk. Is low-fat milk a healthier choice than full-fat? According to a 2017 study by Tognon et al., all-cause mortality is significantly higher among adults who consumed full-fat milk compared to medium- or low-fat milk [15]. Similarly, Talaei et al. found that Iranians who drink whole milk daily are at higher risk of all-cause mortality than their counterparts [16]. Likewise, whole milk intake is predictive of elevated prostate cancer mortality in studies by Lu et al. [17] and Song et al. [18]. Conversely, according to Crichton and Alkerwi, high intake of whole-fat dairy is inversely associated with obesity and abdominal adiposity compared to those with low consumption [19]. Additionally, research by Drehmer et al. shows that full-fat, but not low-fat, dairy is favorably related to the metabolic syndrome in adults [20].

The effects of milk and milk fat on cancer, heart disease, diabetes, and all-cause mortality have been reviewed extensively in the literature. With some investigations showing positive outcomes and others revealing negative, there remains much to learn about the effect of cow's milk on health and disease in adults. The influence of milk fat, particularly low-fat compared to full-fat, also needs clarification.

To date, the influence of milk and milk fat on inflammation, oxidative stress, and cellular longevity has received little attention. Cellular longevity is often indexed objectively by measuring the length of telomeres [21, 22]. Adults with short telomeres tend to have more oxidative stress and chronic disease, including more heart disease, depression, obesity, and cancer, as well as earlier death, than their counterparts [23–25].

Telomeres add stability to and help safeguard chromosomes. Telomeres cap the ends of chromosomes with nucleoproteins. A simple analogy is that telomeres function like the caps that protect the end of shoe laces. Over time, as cells divide, telomeres become progressively and predictably shorter.

Although chronological age is the key factor accounting for the length of telomeres, other things contribute significantly. Research shows that oxidative stress is a critical factor [26, 27]. Moreover, lifestyle plays a major role. For instance, people who smoke have shorter telomeres than nonsmokers [28]. Adults with obesity have shorter telomeres than their counterparts [29], and inactive individuals have shorter telomeres than those who are physically active [30].

Biological aging is also affected by diet. Regular intake of healthy foods like nuts and seeds is associated with longer telomeres [31], whereas consumption of less healthy foods, like processed meats, is related to shorter telomeres [32]. Fiber intake goes hand-in-hand with longer telomeres [33], as does higher vegetable intake [34] and regular fruit consumption [35]. However, consumption of fats and oils is associated with shorter telomeres and increased biological aging [36, 37].

From a more physiological and molecular nutrition perspective, milk is a postnatal endocrine signaling system [38]. Milk consumption encourages mTORC1-mediated anabolism and growth. For example, extended full-fat cow's milk consumption in mice increases energy intake and body weight and reduces insulin signaling in white adipose tissue compared to low-fat milk intake [39]. According to Melnik, to accomplish its mTORC1-activating role, four metabolic messengers are provided by milk: “1. essential branched-chain amino acids, 2. glutamine, 3. palmitic acid, and 4. bioactive exosomal microRNAs…” [38].

Research by Yasuda et al. indicates that unsaturated and saturated fatty acids have opposite effects on podocyte apoptosis by controlling mTORC1 activity via translocation onto lysosomal membranes [40]. Insulin and IGF-1 and essential branched-chain amino acids influence mTORC1 by activation of the kinase AKT pathway. Palmitic acid, the primary saturated fatty acid of milk fat globules, also activates mTORC1 at the lysosome [40]. In short, it appears that repeated mTORC1 activation contributes to endoplasmic reticulum stress, leading to premature aging and disease [38].

Although much is understood about diet, oxidative stress, and cellular longevity, the role of cow's milk consumption, particularly milk fat, remains unclear. To date, the relationship between milk fat intake and telomere length has rarely been studied. Hence, the present study was conducted. Its purpose was to determine the extent cow's milk consumption and the fat content of the milk account for differences in cellular aging, indexed using leukocyte telomere length in 5,834 women and men, representative of the U.S. adult population. A secondary objective was to assess the extent demographic, lifestyle, and other dietary factors influence the milk and telomere relationships. The role of saturated fat intake, a major part of the fat content of cow's milk, was also a significant focus of the investigation.

## 2. Materials and Methods

### 2.1. Sample

For a number of decades, the National Health and Nutrition Examination Survey (NHANES) has been conducted in the United States. NHANES is a government-sponsored research program administered by the National Center for Health Statistics. The purpose of NHANES is to evaluate the health and nutrition status of children and adults in the United States and to track national trends over time using physical examinations, interviews, and laboratory tests.

NHANES recruits participants using a four-stage, probability sampling design [41]. Consequently, if analyzed correctly using individual sample weights, results are generalizable to the U.S. population. Although NHANES data have been collected for many decades, telomere data are only available for four years, 1999-2002. Data containing telomere values have been available for public use since the end of 2014. NHANES data are cross-sectional and free and can be accessed by the public online [41, 42].

During the few years that telomere data were collected by NHANES, only individuals who were at least 20 years old were given the opportunity to give a sample of DNA. Of the 10,291 eligible participants, a total of 7,827 useable DNA samples were obtained. Individuals who were 85 years or older were not included in the present sample because NHANES recorded the age of all these adults as 85 years, truncating their ages, to safeguard privacy.

Each NHANES subject was required to give written informed consent to participate. Moreover, approval to collect the individual data and post it online without identifying information was approved by the Ethics Review Board of the National Center for Health Statistics [43].

### 2.2. Measurements

In this investigation, there were two exposure variables: (1) frequency that cow's milk was consumed and (2) the milk fat content of the milk consumed. The outcome variable was leukocyte telomere length, a marker of biological aging. There were a dozen covariates controlled in the present study (age, gender, race, household size, smoking, body mass index, MET-minutes of total physical activity, alcohol use, grams of protein consumed per kilogram of body weight, percentage of energy derived from dietary fat, grams of dietary fiber consumed per 1000 kilocalories, and percentage of total energy derived from saturated fat.

#### 2.2.1. Milk Consumption

The Diet Behavior and Nutrition section of the NHANES questionnaire was used to collect data on milk consumption. One question focused on frequency of milk consumption during the past 30 days. The specific NHANES question was: “Now I'm going to ask a few questions about milk products. Do not include their use in cooking. In the past 30 days, how often did you have milk to drink or on your cereal? Please include chocolate and other flavored milks as well as hot cocoa made with milk. Do not count small amounts of milk added to coffee or tea.” Possible responses were: Never; Rarely—less than once a week; Sometimes—once a week or more, but less than once a day; or Often—once a day or more. Participants could also indicate that their milk consumption is “Varied.” Only 37 individuals selected the “Varied” option, which is an insufficient subsample to analyze according to NHANES, so the “Varied” group was excluded from the analyses.

Participants who indicated that they drank milk were asked: “What type of milk was it? Was it usually…?” Possible responses were: You drink whole or regular milk; You drink 2% fat milk; You drink 1% fat milk; You drink skim, nonfat, or .5% milk (which includes reconstituted from dry); or You drink another type of milk. Subjects who reported that they drink a milk type other than cow's milk (*n* = 185), such as almond milk and soy milk, were excluded from the study. Individuals who indicated that they do not drink milk were labeled milk abstainers for both the milk frequency question and the milk fat question.

Concurrent validity was shown for both the milk consumption frequency question and the milk fat measure. Specifically, adults reporting that they consumed milk Rarely, Sometimes, or Frequently consumed more saturated fat than adults who never consumed milk. Those reporting that they consumed milk Frequently or Sometimes had higher protein intakes than those indicating that they consumed milk Rarely or Never. Additionally, adults consuming full-fat or 2% milk had higher total dietary fat intakes than milk abstainers and those consuming nonfat milk. Similarly, those who consumed full-fat or 2% milk consumed higher levels of saturated fat than milk abstainers and those drinking 1% milk or nonfat milk.

Information about the thermal processing of milk was not collected by the NHANES questionnaire. Specifically, no data were gathered about milk pasteurization versus ultra-heat-treated (UHT) milk. This is a study limitation because milk-derived miRNAs survive pasteurization, and recent research shows that miRNAs may play a role in telomere length [44].

#### 2.2.2. Telomere Length

According to NHANES [45], “the telomere length assay was performed in the laboratory of Dr. Elizabeth Blackburn at the University of California, San Francisco, using the quantitative polymerase chain reaction method to measure telomere length relative to standard reference DNA (T/S ratio), as described in detail elsewhere [46, 47]. Each sample was assayed 3 times on 3 different days. The samples were assayed on duplicate wells, resulting in 6 data points. Sample plates were assayed in groups of 3 plates, and no 2 plates were grouped together more than once. Each assay plate contained 96 control wells with 8 control DNA samples. Assay runs with 8 or more invalid control wells were excluded from further analysis (<1% of runs). Control DNA values were used to normalize between-run variability. Runs with more than 4 control DNA values falling outside 2.5 standard deviations from the mean for all assay runs were excluded from further analysis (<6% of runs). For each sample, any potential outliers were identified and excluded from the calculations (<2% of samples). The mean and standard deviation of the T/S ratio were then calculated normally. The interassay coefficient of variation was 6.5%” [45]. To convert average *T*/*S* ratios to base pairs, the following formula was applied: 3274 + 2413 × (*T*/*S*).

#### 2.2.3. Covariates

In the present investigation, age, gender, race, and household size were used as demographic covariates. For race/ethnicity, five NHANES categories were used: Non-Hispanic White, Non-Hispanic Black, Mexican American, Other Race including Multi-Racial, and Other Hispanic. Household size was defined as the number of individuals living in the house or apartment. The maximum number of individuals counted by NHANES was seven.

Smoking (pack-years), body mass index (BMI), total physical activity (MET-minutes of activity per week), and alcohol use were employed as lifestyle covariates. Pack-years of smoking was calculated by multiplying the number of cigarettes smoked per day by the number of years the person smoked, divided by 20, the number of cigarettes in a pack. BMI was used as an index of body weight independent of height. BMI was calculated by dividing weight in kg by height in meters, squared (kg/m^2^).

Total physical activity (PA) was indexed using MET-minutes of activity. A MET is a metabolic equivalent. From a list of 48 physical activities, including tennis, walking, gardening, hiking, swimming, bicycling, and many more, participants reported which, if any, they participated in during the past 30 days. Using NHANES descriptions, subjects indicated if their participation was vigorous or moderate. Using the compendium of physical activity, a MET value for each activity was assigned [48]. A total activity score was calculated by summing the MET-minutes of each activity and converting the score to a weekly value.

Alcohol use was defined by NHANES using three categories: heavy drinkers, moderate drinkers, and abstainers. Heavy drinkers were women who had ≥2 alcoholic beverages per day or men who consumed ≥3 drinks per day. Moderate drinkers were women who had >0 and <2 drinks per day or men who reported >0 and <3 drinks per day.

Because adults who drink whole or full-fat milk compared to those who choose nonfat milk may differ in their consumption of other dietary factors, several dietary measures were assessed and used as covariates: grams of protein per kilogram of body weight, percentage of energy from dietary fat, grams of dietary fiber per 1000 kilocalories, and the percentage of total energy from saturated fat. These dietary covariates were measured using a 24-hour recall. Because day-of-the-week influences dietary intake and was not randomized by NHANES, a specific set of sample weights was employed by NHANES to accommodate the 24-hour recall characteristics. In general, the 24-hour recall sample weights resulted in larger variance estimates than the MEC (mobile examination center) sample weights [49].

### 2.3. Data Analysis

An individual sample weight was assigned to each participant by NHANES. Each sample weight signified the number of people in the U.S. represented by the sample person. When analyses are conducted using the sample weights, unbiased national estimates are produced. Because sample weights were used in the analyses, the results can be generalized to all noninstitutionalized, civilian adults in the U.S.

To describe the data, means (±SE) were calculated for continuous variables and frequencies were computed for categorical variables. To determine the extent that mean telomere values differed across categories of milk intake frequency (i.e., Never, Rarely, Sometimes, and Often), analysis of variance (ANOVA) was performed using regression analysis. Similarly, telomere means were compared across categories based on the level of milk fat consumption (abstainer, full-fat, 2%, 1%, and nonfat). In an additional regression analysis to assess the extent of the linear relationship between milk fat intake and biological aging, the fat content of the milk consumed was treated as a continuous variable. To evaluate the influence of 12 covariates, adjustments were made employing partial correlation and the SAS SurveyReg procedure.

Analyses were also conducted to test the presence of effect modification for two conditions. First, the relationship between milk fat intake and telomere length was evaluated with the milk consumption frequency variable delimited to adults reporting that they consumed milk “Often,” the highest category. Another analysis was performed with the milk intake frequency variable delimited to adults who consumed milk “Sometimes,” and another analysis was conducted with the sample delimited to those consuming milk “Rarely.” Second, the effect modification was tested across tertiles of total saturated fat intake. Specifically, the association between milk fat intake and biological aging was evaluated in those who consumed low levels of total saturated fat (tertile 1), moderated levels (tertile 2), and high levels (tertile 3), separately.

The telomere variable distribution was not normal, so the telomere values were log-transformed. To assist with interpretation of the results, nontransformed means are reported.

Many scientists assume that statistical power associated with NHANES investigations is very high because of the large sample size. However, because NHANES uses a four-stage sampling process with nesting, all the analyses were conducted with 29 degrees of freedom in the denominator, even the analyses involving the effect modification. The 29 degrees of freedom resulted from subtracting the 28 strata from the 57 clusters.

SAS version 9.4 (SAS Institute, Inc., Cary, NC) was used to conduct the statistical analyses. Statistical significance was accepted when alpha was less than 0.05 and all *P* values were two-sided.

## 3. Results

A total of 3,072 women and 2,762 men were included in the study (*n* = 5,834). Prevalence and weighted percentages for the frequency of milk consumption and milk fat content variables, along with each of the categorical covariates, are displayed in Table 1. For the continuous variables, average age (±SE) of the sample was 46.4 (±0.5) years. Mean MET-minutes of total physical activity was 130.3 (±9.6) per week, and average telomere length was 5836.7 (±39.4) base pairs. The findings of the present investigation are generalizable to the noninstitutionalized adult population of the United States because participants were randomly selected and sample weights were employed as part of the analyses.

As shown in Table 1, nearly half of U.S. adults consumed milk daily and another 1 in 4 consumed milk at least weekly, but not daily. Approximately 30% of U.S. adults reported consuming full-fat milk and another 30% drank 2% milk. Combined, about 60% of U.S. adults reported drinking high-fat milk. On the other hand, 10% reported consuming 1% milk and another 17% indicated they drank nonfat milk. Combined, about 27% reported drinking low-fat milk. About 13% reported never consuming cow's milk.

The weighted % column shows the distribution of subjects after the NHANES sample weights were applied. The weighted % values are more meaningful than the number of subjects because they take into account the sample weights and reflect the percentage of the U.S. population that practice the behavior.

These results are similar to those from the most recent NHANES data available. Recent NHANES data show that approximately 36% report consuming 2% milk and another 21% drink full-fat milk. Approximately 13% drink nonfat milk and 10.5% indicate 1% milk. Hence, in recent years, 57% drink high-fat milk and 23.5% report drinking low-fat milk. These recent NHANES milk data could not be used in the present study because telomere data were not available during these years.

An inverse, linear relationship was detected between chronological age and telomere length. Leukocyte telomeres were 15.3 base pairs shorter for each additional year of age (*F* = 623.3, *P* < 0001). The Pearson correlation was inverse and significant (*r* = ‐0.43, *P* < 0.0001). Beyond the linear association, telomere length was not related to age-squared (*F* = 0.3, *P* = 0.6020).

Table 2 shows that the mean length of telomeres did not differ across levels of milk consumption frequency in U.S. adults. Telomere length differences were not significant after adjusting for the demographic, lifestyle, and/or dietary covariates.

Table 3 indicates that mean telomere lengths differed across levels of milk fat consumption, after controlling for the demographic covariates, the demographic and lifestyle covariates, and the dietary covariates, in addition to the other covariates. After adjusting for all the covariates, adults who consumed full-fat or 2% milk had shorter telomeres than those who consumed nonfat or 1% milk. The difference in telomere length was 145 base pairs. Similarly, milk abstainers had shorter telomeres than those consuming nonfat milk or 1% milk. Consumers of nonfat milk had telomeres that were 115 base pairs longer than those who did not drink cow's milk, on average. Telomere length did not differ among those who consumed full-fat milk and 2% milk or who were milk abstainers. Adults who consumed nonfat or 1% milk had statistically equal mean telomere lengths.

With milk fat intake treated as a continuous variable and milk abstainers held out of the analysis, milk fat content was linearly and inversely related to telomere length, after adjusting for differences in the demographic variables (*F* = 9.7, *P* = 0.0042) and the demographic and lifestyle measures (*F* = 8.7, *P* = 0.0063) and after controlling for the demographic, lifestyle, and dietary variables together (*F* = 8.6, *P* = 0.0066). With all the covariates controlled, for each increment of 1 percentage point of milk fat (e.g., 1% to 2% milk fat, or 2% to 3%), telomeres were 69 base pairs shorter, on average.

The effect modification was tested with subjects stratified according to milk consumption frequency levels. Results showed that the relationship between milk fat content and telomere length was significant when the sample was delimited to adults who consumed milk “Often” (daily or more) or “Sometimes” (at least weekly, but not daily), as displayed in Table 4. However, when the sample was delimited to adults who consumed milk “Rarely,” there was no association between milk fat content and biological aging.

The effect modification was also evaluated with participants separated into tertiles based on total saturated fat intake (percent of total energy derived from saturated fat). With the sample delimited to adults with high levels of total saturated fat intake (tertile 3), the relationships between milk fat intake and telomere length were stronger for Model 2 (*F* = 4.5, *P* = 0.0059) and Model 3 (*F* = 6.2, *P* = 0.0010), while Model 1 (*F* = 4.1, *P* = 0.0097) was similar to the association shown in Table 3, based on the total sample. With the sample confined to adults with only moderate intakes of total saturated fat (tertile 2), the relationships between milk fat consumption and telomere length were weaker, but remained significant, for Model 1 (*F* = 3.4, *P* = 0.0209), Model 2 (*F* = 3.2, *P* = 0.0276), and Model 3 (*F* = 3.5, *P* = 0.0191), compared to models shown in Table 3 based on the total sample. With the sample restricted to U.S. adults who only consumed low levels of total saturated fat (tertile 1), none of the models were statistically significant.

Table 5 displays the extent the dietary covariates (i.e., protein, total dietary fat, fiber, and saturated fat) differed across the five milk fat content categories. Adjustments were made for differences in the demographic covariates. Results showed that all the relationships were statistically significant (Table 5). Adults who did not drink cow's milk consumed less protein than any of the other groups (*F* = 12.2, *P* < 0.0001). Also, nonfat milk drinkers consumed less fat than any of the other groups. On the other hand, adults who drank full-fat milk or 2% consumed more dietary fat than abstainers and nonfat milk drinkers (*F* = 28.9, *P* < 0.0001). Moreover, milk abstainers and those who drank nonfat milk consumed less saturated fat than adults who drank full-fat, 2%, or 1% milk (*F* = 49.8, *P* < 0.0001). Lastly, nonfat milk drinkers consumed more dietary fiber than any of the other milk fat groups, and those who drank full-fat milk ate less fiber than any of the other milk fat groups (*F* = 64.1, *P* < 0.0001), as shown in Table 5.

## 4. Discussion

The primary purpose of this study was to investigate the relationship between leukocyte telomere length, an objective, biological marker of cellular aging, and two outcome variables, milk intake frequency and the milk fat content typically consumed by U.S. adults. The data were part of the National Health and Nutrition Examination Survey (NHANES). To date, this association has been studied rarely. An ancillary aim was to evaluate the influence of multiple demographic, lifestyle, and dietary covariates on the associations between milk consumption and telomere length. Another objective was to test the presence of the effect modification by examining the association between the amount of milk fat typically consumed and telomere length, with the sample delimited to individual levels of milk consumption frequency (i.e., Often, Sometimes, and Rarely). A second effect modification was evaluated by examining the relationship between milk fat intake and cellular aging within three distinct categories of saturated fat intake, based on tertiles (i.e., low, moderate, and high).

A number of meaningful findings were uncovered in the present study. First, adults who drink full-fat or 2% milk (i.e., high-fat milk) have significantly and meaningfully shorter telomeres than adults who drink nonfat or 1% milk (i.e., low-fat milk). Milk abstainers also have shorter telomeres than adults who consumed low-fat milk. Second, the relationship between milk fat content and telomere length is the strongest when the sample is delimited to adults who drink milk “Often,” at least once per day. There is no association between milk fat intake and telomere length in adults that drink milk “Rarely,” less than weekly. Third, frequency of milk consumption is not related to telomere length in U.S. adults. Fourth, adjusting for differences in demographic, lifestyle, and dietary covariates has little influence on the relationship between milk fat intake and biological aging. Fifth, there is no relationship between milk fat intake and telomere length among adults who consumed low levels of total saturated fat (tertile 1), but it is strong among adults who consumed moderate or high levels of total saturated fat.

Current Dietary Guidelines for Americans (2015–2020) [50] encourage adults to consume low-fat milk, including nonfat and 1% milk. The guidelines discourage the consumption of high-fat milk, specifically 2% and whole milk. As shown in Table 1, only 17% of U.S. adults consumed nonfat milk and 10% drank 1%, a total of 27%. On the other hand, approximately 30% drank 2% milk and another 30% indicated that they consumed full-fat (whole) milk (Table 1). In short, it appears that more than twice the U.S. adults (60% vs. 27%) choose high-fat milk over low-fat milk, contrary to current U.S. dietary recommendations and findings of the present investigation [50].

Current dietary guidelines were not in effect during the time NHANES data were collected for the present investigation. Using the most recent data available from NHANES shows that in recent years, 57% of U.S. adults report drinking high-fat milk and 23.5% report consuming low-fat milk. Obviously, milk fat intake remains elevated and has not changed much over time. Clearly, there is a substantial gap between recommended low-fat milk consumption and practices of the past and recent years. It appears that a large percentage of U.S. adults continue to consume high-fat milk, which is associated with unfavorable biological aging according to the present study.

With both the milk fat and telomere length variables treated as continuous measures, the sample delimited to milk consumers, and all the covariates controlled statistically, there was a significant inverse, linear association between milk fat intake and telomere length. The more milk fat subjects consumed, the shorter their telomeres tended to be. For each 1 percentage point increase in milk fat consumed, telomeres were 69 base pairs shorter, on average. Because each additional year of chronological age in the present sample was associated with telomeres that were 15.3 base pairs shorter, on average, it follows that a 1 percentage point increase in milk fat (e.g., 1% to 2%) is associated with more than 4 additional years of biological aging (69 base pairs ÷ 15.3 = 4.5).

With milk fat intake treated as a categorical variable (Table 3), there was a substantial biological aging difference between high-fat milk consumers (2% and full-fat) and low-fat milk consumers (1% and nonfat). The average difference was 145 telomere base pairs between the two extremes, after adjusting for all the covariates. On average, telomeres were 15.3 base pairs shorter for each year of chronological age in the sample, so a mean difference of 145 base pairs represents a very large difference in cellular aging between high-fat and low-fat milk drinkers.

The effect modification was evaluated by examining the relationship between milk fat intake and telomere length within each level of milk intake frequency (i.e., Often, Sometimes, and Rarely; see Table 4). With the sample delimited to only those reporting that they consumed milk “Often” (i.e., at least daily), the association was the strongest. Within those reporting that they drink milk “Sometimes” (i.e., at least weekly, but not daily), the association remained significant, but was weaker. Delimited to those claiming to drink milk “Rarely” (i.e., less than weekly), there was no relationship between milk fat intake and telomere length. Given these systematic findings, it appears that the more frequently milk is consumed, the stronger the connection between milk fat and cellular aging. This finding adds credence to the milk fat and aging association found in the present study.

In the current study, those reporting regular high-fat milk intake (full-fat or 2%) had substantially shorter telomeres, on average, than those consuming low-fat milk (1% or nonfat), indicating that high-fat milk intake goes hand-in-hand with increased cellular aging. However, individual foods are seldom eaten in isolation. Results displayed in Table 5 show this. Adults who preferred nonfat milk consumed less dietary fat and less saturated fat, as well as more dietary fiber, than their counterparts. Some of the dietary differences revealed among the milk fat groups were likely due to the milk composition differences that exist across the milk fat categories. However, it is possible that other dietary differences account for some of the biological aging differences among the milk fat categories.

The present investigation revealed that high-fat milk drinkers consume significantly more saturated fat than their counterparts (Table 5). This is logical since milk fat is 60-65% saturated. Given the bulk of the literature, it is possible that some of the increase in biological aging in U.S. women and men stems from the higher saturated fat intake among high-fat milk consumers. This idea is supported by the effect modification results uncovered when the milk fat and telomere length association was evaluated within tertiles of saturated fat intake. When the sample was delimited to adults reporting only high saturated fat intake (tertile 3), the milk fat and telomere relationship was stronger than when the total sample was used. However, when the sample was restricted to adults reporting only low saturated fat consumption (tertile 1), there was no relationship between milk fat intake and telomere length. Apparently, if total saturated fat intake is low, the amount of milk fat consumed matters little and does not relate to biological aging. However, when total saturated fat intake is high (or moderate), milk fat consumption plays a significant role in cellular aging and telomere length.

Why was the amount of milk fat typically consumed by U.S. adults related significantly to telomere length in the present study? Although the exact mechanism is unknown, the fact that telomere length and genomic stability are highly related to oxidative stress and inflammation is frequently noted in the literature [51–54]. In a review paper by Rocha et al., the authors explain how saturated fats trigger inflammatory pathways, alter gut microbiota, and lead to increased oxidative stress [55]. Animal research also indicates that high-fat and high saturated fat diets induce inflammation and oxidative stress [56]. Similarly, Marin et al. showed that reactive oxidative species are significantly higher when humans are fed a saturated fatty acid diet, with butter providing most of the fat, compared to a Mediterranean diet, with olive oil serving as the main source of fat [57]. In short, it appears that the milk fat and cellular aging association identified in this investigation was probably due, in part, to increased inflammation and oxidative stress caused by increased consumption of saturated fat.

For the prevention of premature aging and death, the type of dietary fat is probably more important than fat quantity [58]. According to a 2017 Presidential Advisory from the American Heart Association, “Taking into consideration the totality of the scientific evidence, satisfying rigorous criteria for causality, we conclude strongly that lowering intake of saturated fat and replacing it with unsaturated fats, especially polyunsaturated fats, will lower the incidence of CVD” [59]. Moreover, in a prospective study of more than 7000 adults, Guasch-Ferré et al. found that higher levels of saturated fat were associated with an 81% higher risk of CVD [60]. Others have shown similar findings for saturated fat, especially when saturated fats are replaced with unsaturated fats [61–64]. However, not all support the value of reducing saturated fat intake to reduce oxidative stress, chronic disease, and mortality [65].

In a telomere study limited to postmenopausal women, Song et al. showed that consumption of small to medium chain fatty acids is inversely related to telomere length [36]. Note, milk fat includes a significant amount of small to medium chain fatty acids. Other than lauric acid, all other small to medium chain fatty acids were inversely associated with telomere length in the postmenopausal women. Additionally, when divided into quartiles, the percent of energy from saturated fatty acids showed a significant relationship with cellular aging. As the percent of energy from saturated fats increased, cellular aging increased. Monounsaturated fats and polyunsaturated fats were not associated with cellular aging in the investigation by Song et al. [36].

Palmitic acid, the main saturated fatty acid of milk fat globules, is a long-chain fat. According to Fatima et al. [66], palmitic acid is an intracellular signaling molecule involved significantly in disease development. The signaling that increases cellular concentrations of palmitic acid can lead to programmed cell death by multiple mechanisms [67]. Palmitic acid affects the expression of 162 genes in liver cells and is a regulator of genes not linked to the liver or fat metabolism [68]. Delivered by milk consumption, palmitic acid promotes mTORC1-mediated activation and growth, contributing to endoplasmic reticulum stress and premature aging [40]. Furthermore, full-fat milk carries at least three other messengers, besides palmitic acid, that promote mTORC1-mediated activation: essential branched-chain amino acids, glutamine, and bioactive exosomal microRNAs [38].

High levels of palmitic acid are poisonous and can cause apoptosis without the involvement of reactive oxygen species [69]. Small changes in palmitic acid levels can lead to substantial biological changes and cell death [70]. As stated by Ford, saturated fatty acids, particularly palmitic acid, are the key link between cell division, cancer, and senescence in cellular and whole organism aging [69].

There is strong support linking obesity, prostate and breast cancer, type 2 diabetes, and neurodegenerative diseases with increased expression of the FTO (obesity) gene. Zhou et al. discuss the role of the FTO gene in the regulation of methylation status as possible regulators of genetic aging [71, 72]. Moreover, SNPs (single-nucleotide polymorphisms) within intron 1 of the FTO gene are linked with increased FTO expression, increased body weight, obesity, and type 2 diabetes [73]. Translational evidence also links milk signaling with FTO-activated transcription of the milk consumer [73].

Cow's milk delivers significant quantities of exosomal miRNA-29s that reach the systemic circulation and target mRNAs of the milk drinker. By way of DNA methyltransferases (DNMT) suppression, milk exosomal miRNA-29s may decrease FTO methylation, thereby epigenetically increasing FTO expression in the milk consumer [73]. According to Melnik, “Maximization of lactation performance by veterinary medicine with enhanced miRNA-29s and FTO expression associated with increased exosomal miRNA-29 and FTO mRNA transfer to the milk consumer may represent key epigenetic mechanisms promoting FTO/mTORC1-mediated diseases of civilization” [73]. Clearly, these factors could account for the shorter telomeres and increased aging found in adults with higher intakes of full-fat milk compared to their counterparts.

The present investigation was not without limitations. First, subjects who consumed high-fat milk may be different from those who drink low-fat milk. High-fat milk consumers may have lifestyles that are less healthy than low-fat milk drinkers. Since this possibility was recognized before the onset of the investigation, statistical adjustments were made for a dozen potential confounders. Statistical analyses determined that these variables had little influence on the milk fat and telomere relationship. Nevertheless, other variables could explain some of the relationship between milk fat intake and telomere length identified in the present investigation. Second, NHANES collects data on individuals cross-sectionally. Cross-sectional data cannot be used to infer cause-and-effect. Causation is a possibility, and the present findings warrant additional research in this area, but the scope of this study does not allow causal conclusions. Third, NHANES did not collect information about the thermal process employed to treat the milk consumed by participants. No data were gathered about milk pasteurization versus ultra-heat-treated (UHT) milk. Because milk-derived miRNAs survive pasteurization, and recent research shows that miRNAs may play a role in telomere length and aging, this was a study limitation [44].

There were also multiple strengths associated with the present investigation. For example, the multiracial sample included almost 6000 men and women who were randomly selected from the U.S. population. Therefore, the results are generalizable to all noninstitutionalized, civilian adults in the United States. Additionally, a highly respected lab performed the measurements needed to determine the length of telomeres. Moreover, the relationship between chronological age and telomere length was strong and meaningful, as it should be, adding credence to the telomere measurement. Fourth, a dozen demographic, lifestyle, and dietary covariates were controlled statistically, reducing their influence on the association between milk consumption and telomere length. Finally, multiple effect modification models were evaluated showing that as the frequency of milk consumption increased, the stronger the link was between milk fat and telomere length and that there was no relationship between milk fat and telomere length when total saturated fat intake was low, but the association was strong when total saturated fat intake was high.

## 5. Conclusion

Milk consumption in adults, particularly high-fat milk intake, is a controversial topic with mixed findings in the literature. The key finding of the present study was that U.S. adults who typically drink high-fat milk have substantially shorter telomeres than those who drink low-fat milk. In other words, high-fat milk drinkers have more advanced biological aging than low-fat milk consumers. The aging difference between those reporting full-fat or whole milk consumption compared to those who drink nonfat milk was 145 telomere base pairs, representing years of increased biological aging. The effect modification testing showed that the relationship may be partly due to the differences in saturated fat intake across the milk fat categories. However, besides saturated fat, particularly palmitic acid, full-fat milk also delivers essential branched-chain amino acids, glutamine, and bioactive exosomal microRNAs, which promote mTORC1-mediated activation and may contribute to endoplasmic reticulum stress and accelerated aging. Overall, the present investigation highlights the potential cellular aging disadvantage associated with U.S. adults consuming high-fat milk. The results support the latest Dietary Guidelines for Americans (2015–2020) which recommend consumption of low-fat milk and discourage intake of high-fat milk as part of a healthy diet.

## Figures and Tables

**Table 1:** Descriptive characteristics of the sample (*n* = 5,834).

Variable	*N*	Weighted %	SE
Gender			
Men	2762	47.4	0.5
Women	3072	52.6	0.5
Race			
Non-Hispanic White	2881	72.5	2.1
Non-Hispanic Black	1066	10.1	1.3
Mexican American	1446	7.5	0.9
Other Race	148	3.4	0.6
Other Hispanic	293	6.5	1.6
Household size			
1	716	11.6	0.6
2	1781	32.0	1.3
3	1065	19.3	1.0
4	949	19.1	1.2
5	616	9.6	0.7
6	273	3.5	0.6
7 or more	434	5.0	0.6
Body mass index			
Underweight	85	1.8	0.2
Normal weight	1598	30.2	0.8
Overweight	2059	33.9	1.2
Obese	1910	31.4	1.1
Missing	182	2.8	0.3
Alcohol use			
Abstainer	2364	36.0	2.6
Moderate drinker	1736	31.4	1.8
Heavy drinker	1734	32.6	1.1
Milk intake frequency^∗^			
Never	814	13.3	0.7
Rarely	761	13.2	0.7
Sometimes	1451	25.0	0.7
Often	2808	48.5	1.1
Milk fat consumed			
Milk abstainers	814	13.3	0.7
Full-fat	2094	29.8	1.4
2% milk	1650	29.9	1.3
1% milk	505	10.0	1.1
Nonfat milk	771	17.0	1.2

^∗^For the milk intake frequency variable, adults reporting “Never” did not consume milk; “Rarely,” consumed milk < once per week; “Sometimes,” consumed milk > once per week, but < once per day; and “Often,” consumed milk at least once per day. SE is standard error of the weighted percentage.

**Table 2:** Differences in mean telomere length (base pairs) by frequency of milk consumption in U.S. women and men, after adjusting for the covariates (*n* = 5,834).

	Frequency of milk consumption^∗^					
Model	Never (mean ± SE)	Rarely (mean ± SE)	Sometimes (mean ± SE)	Often (mean ± SE)	*F*	*P*
Model 1	5814 ± 39	5805 ± 35	5845 ± 42	5811 ± 38	0.8	0.4856
Model 2	5830 ± 40	5836 ± 37	5865 ± 44	5831 ± 40	0.9	0.4733
Model 3	5789 ± 43	5808 ± 44	5822 ± 48	5814 ± 45	0.5	0.7129

SE is the standard error of the mean. ^∗^The four levels of milk consumption frequency were as follows: Never: participants who never consumed milk (*n* = 814, 14.0%); Rarely: adults who consumed milk < once per week (*n* = 761, 13.0%); Sometimes: consumed milk at least once per week, but < once per day (*n* = 1451, 24.9%); and Often: consumed milk once per day or more frequently (*n* = 2808, 48.1%). The number of subjects in each category above does not take into account the sample weights used for each participant. However, the percentage (%) value following the sample size shows the proportion of subjects in the category with the NHANES sample weights applied. The percentage (%) values are more meaningful than the number of subjects (*n*) because the % values represent the proportion of the U.S. adult population that fit the category. Model 1 compares telomere base pairs across each of the four levels of milk consumption frequency, after adjusting for the demographic covariates. Model 2 controls for the demographic and lifestyle covariates. Model 3 controls for the demographic, lifestyle, and dietary covariates.

**Table 3:** Differences in mean telomere length (base pairs) by level of milk fat content consumed by U.S. men and women, after adjusting for the covariates (*n* = 5,834).

	Milk fat content typically consumed						
Model	Milk abstainer (mean ± SE)	Full-fat (mean ± SE)	2% (mean ± SE)	1% (mean ± SE)	Nonfat (mean ± SE)	*F*	*P*
Model 1	5831^a^ ± 39	5771^a^ ± 43	5822^a^ ± 46	5955^b^ ± 67	5926^b^ ± 42	4.5	0.0061
Model 2	5848^a^ ± 40	5798^a^ ± 43	5847^a^ ± 51	5975^b^ ± 74	5942^b^ ± 50	3.3	0.0232
Model 3	5814^a^ ± 44	5784^a^ ± 47	5801^a^ ± 50	5963^b^ ± 85	5929^b^ ± 54	4.1	0.0093

The five levels of milk fat consumption were defined as follows: abstainer: participants who never consumed milk (*n* = 814, 14.0%); full-fat: adults who typically consumed whole or full-fat milk (*n* = 2094, 35.9%); 2%: subjects who usually consumed 2% milk (*n* = 1650, 28.3%); 1%: individuals who typically consumed 1% milk (*n* = 505, 8.7%); and nonfat: participants who typically consumed nonfat, skim, or 0.5% milk (*n* = 771, 13.2%). The number of subjects in each category does not take into account the sample weights assigned to each subject. However, the percentage (%) following sample size shows the proportion of subjects in the milk fat category with the NHANES sample weights applied. SE is the standard error of the mean. ^a,b^Means on the same row with the same superscript letter are not statistically different (*P* > 0.05). Model 1 compares telomere means, after adjusting for the demographic covariates. In Model 1, the mean difference in telomere length between milk abstainers and those who consumed 1% milk was *P* = 0.0564. Model 2 compares telomere means, after controlling for the demographic covariates and the lifestyle covariates. In Model 2, the difference in telomere length between abstainers and those who consumed 1% milk was *P* = 0.0589. Model 3 compares telomere means, after adjusting for the demographic, lifestyle, and dietary covariates.

**Table 4:** Differences in mean telomere lengths (base pairs) by level of milk fat content, after adjusting for all the covariates, with the sample delimited to one milk consumption frequency group at a time.

	Milk fat content typically consumed					
Milk frequency^∗^	Full-fat (mean ± SE)	2% (mean ± SE)	1% (mean ± SE)	Nonfat (mean ± SE)	*F*	*P*
Often (*n* = 2,662)	5766^a^ ± 63	5754^a^ ± 59	5944^b^ ± 95	5902^b^ ± 58	5.4	0.0044
Sometimes (*n* = 1,378)	5798^a^ ± 43	5847^a,b^ ± 51	5975^b^ ± 74	5942^b^ ± 50	3.0	0.0447
Rarely (*n* = 707)	5605^a^ ± 61	5622^a^ ± 62	5722^a^ ± 160	5662^a^ ± 119	0.2	0.8936

^a,b^Means on the same row with the same superscript letter are not significantly different. SE is the standard error of the mean. ^∗^For each row, the sample is delimited to the milk consumption frequency category listed in the first column. For the first row (Often), the sample sizes for full-fat, 2%, 1%, and nonfat were *n* = 935 (28.9%), *n* = 914 (34.3%), *n* = 302 (12.7%), and *n* = 511 (24.1%), respectively. For the second row (Sometimes), the sample sizes were *n* = 647 (37.3%), *n* = 434 (34.5%), *n* = 129 (11.8%), and *n* = 168 (16.4%), respectively. For the third row (Rarely), the sample sizes were *n* = 376 (46.8%), *n* = 221 (35.1%), *n* = 44 (7.0%), and *n* = 66 (11.2%), respectively. The sample size percentages were derived using individual sample weights and therefore represent the U.S. adult population. The sample size numbers do not. Means on each row were adjusted for differences in all the covariates.

**Table 5:** Mean differences in the dietary covariates by level of milk fat consumed by U.S. adults.

	Level of milk fat typically consumed						
Covariate	Abstainer (mean ± SE)	Full-fat (mean ± SE)	2% (mean ± SE)	1% (mean ± SE)	Nonfat (mean ± SE)	*F*	*P*
Protein (g/kg)	0.93^a^ ± 0.03	1.04^b,c^ ± 0.02	0.99^b^ ± 0.02	1.02^b,c^ ± 0.04	1.07^c^ ± 0.03	12.2	<0.0001
Fat (% kcal)	31.2^a^ ± 0.82	32.6^b^ ± 0.60	32.8^b^ ± 0.76	32.1^a,b^ ± 0.73	29.5^c^ ± 0.75	28.9	<0.0001
Sat. fat (% kcal)	9.1^a^ ± 0.26	10.8^b^ ± 0.23	10.5^b^ ± 0.28	10.1^c^ ± 0.29	8.8^a^ ± 0.30	49.8	<0.0001
Fiber (g/1000 kcal)	8.6^a^ ± 0.26	7.1^b^ ± 0.22	8.0^c^ ± 0.30	9.0^a^ ± 0.25	9.9^d^ ± 0.27	64.1	<0.0001

SE is the standard error of the mean. Protein (g/kg) refers to grams of protein intake per kilogram body weight. Fat (% kcal) represents the percentage of total energy derived from dietary fat. Sat. Fat (% kcal) refers to the percentage of total energy derived from saturated fat. Fiber (g/1000 kcal) represents the number of grams of fiber consumed per day per 1000 kcal. The nonfat milk category also included skim milk and 0.5% milk. Means on each row were adjusted for differences in the demographic variables. ^a,b,c,d^Means on the same row with the same superscript letter are not statistically different (*P* > 0.05). On the row for fiber intake, the mean difference between abstainers and adults who drank 2% milk was borderline significant (*P* = 0.0857). On the row for fat (% kcal), the difference between milk abstainers and those drinking nonfat milk was borderline significant (*P* = 0.0920). The five levels of milk fat consumption were defined as follows: abstainer: participants who never consumed milk (*n* = 814, 14.0%); full-fat: adults who consumed full-fat milk (*n* = 2094, 35.9%); 2%: subjects who usually consumed 2% milk (*n* = 1650, 28.3%); 1%: individuals who consumed 1% milk (*n* = 505, 8.7%); and nonfat: participants who typically consumed nonfat, skim, or 0.5% milk (*n* = 771, 13.2%). The number of subjects in each category above does not take into account the sample weights assigned to each subject. However, the percentage (%) following the sample size shows the proportion of subjects in the milk fat category with the NHANES sample weights applied. The % values are more meaningful than the number of subjects (*n*) because the percentages represent the proportion of the U.S. adult population that fall within each milk fat category.

## Data Availability

The U.S. National Center for Health Statistics (NCHS), which is part of the U.S. Centers for Disease Control and Prevention, compiles statistical information to help guide policies to improve the health of Americans. The NCHS supplied the National Health and Nutrition Examination Survey (NHANES) data used in the present study. The NHANES data and related documentation are free to the public and available at https://wwwn.cdc.gov/nchs/nhanes/Default.aspx.
